# A Computational Model of Lipopolysaccharide-Induced Nuclear Factor Kappa B Activation: A Key Signalling Pathway in Infection-Induced Preterm Labour

**DOI:** 10.1371/journal.pone.0070180

**Published:** 2013-07-30

**Authors:** Gemma C. Sharp, Hongwu Ma, Philippa T. K. Saunders, Jane E. Norman

**Affiliations:** 1 Medical Research Council (MRC) Centre for Reproductive Health, University of Edinburgh, Edinburgh, United Kingdom; 2 Computational Systems Biology, School of Informatics, University of Edinburgh, Edinburgh, United Kingdom; 3 Key Laboratory of Systems Microbial Biotechnology, Tianjin Institute of Industrial Biotechnology, Chinese Academy of Sciences, Tianjin, P.R. China; 4 Medical Research Council (MRC) Centre for Reproductive Health and Tommy’s Centre for Maternal and Fetal Health, University of Edinburgh, Edinburgh, United Kingdom; John Hunter Hospital, Australia

## Abstract

Preterm birth is the single biggest cause of significant neonatal morbidity and mortality, and the incidence is rising. Development of new therapies to treat and prevent preterm labour is seriously hampered by incomplete understanding of the molecular mechanisms that initiate labour at term and preterm. Computational modelling provides a new opportunity to improve this understanding. It is a useful tool in (i) identifying gaps in knowledge and informing future research, and (ii) providing the basis for an *in silico* model of parturition in which novel drugs to prevent or treat preterm labour can be “tested”. Despite their merits, computational models are rarely used to study the molecular events initiating labour. Here, we present the first attempt to generate a dynamic kinetic model that has relevance to the molecular mechanisms of preterm labour. Using published data, we model an important candidate signalling pathway in infection-induced preterm labour: that of lipopolysaccharide (LPS) -induced activation of Nuclear Factor kappa B. This is the first model of this pathway to explicitly include molecular interactions upstream of Nuclear Factor kappa B activation. We produced a formalised graphical depiction of the pathway and built a kinetic model based on ordinary differential equations. The kinetic model accurately reproduced published *in vitro* time course plots of Lipopolysaccharide-induced Nuclear Factor kappa B activation in mouse embryo fibroblasts. In this preliminary work we have provided proof of concept that it is possible to build computational models of signalling pathways that are relevant to the regulation of labour, and suggest that models that are validated with wet-lab experiments have the potential to greatly benefit the field.

## Introduction

Our limited understanding of the molecular mechanisms associated with the onset of labour in women at term makes it difficult to pinpoint ‘what goes wrong’ when women go into labour too early. Improving our understanding of this is a major priority because preterm labour induced preterm birth is the single biggest cause of significant neonatal morbidity and mortality [1]–[3]. Rates of preterm birth are rising, and even “perfect” application of current therapies will reduce absolute rates of preterm birth by less than 0.5% [4]. We and others have argued that development of new therapies for preterm labour prevention is seriously hampered by incomplete understanding of the molecular mechanisms that initiate labour at term and preterm [5]. Additionally, attempts to improve our understanding of parturition are often restricted by the inaccessibility of human gestational tissues to study during pregnancy, and by the lack of fully informative animal models.

The novel paradigm of “systems biology” provides a promising opportunity to overcome these restrictions and improve our understanding of the key molecular pathways that initiate labour in women [6]. Systems biology provides useful strategies to integrate the complex interactions within biological systems through building computational models. Such models can be used to develop comprehensive *in silico* reproductions of “pregnant” tissues that demonstrate emergent properties [7]. Computational models of the molecular mechanisms initiating parturition could (i) identify gaps in knowledge where additional wet lab experiments are required, and (ii) provide the basis for an *in silico* model of parturition for “testing” novel drugs to treat or prevent preterm labour.

Computational modelling is a major growth area in biomedical research, but has only rarely been applied to pregnancy physiology or pathology. There is only one report of a computational model to study the molecular events initiating labour [8]. This model by Equils et al. uses published data to model the immune-endocrine interactions in a uterine smooth muscle cell with an increase in the ratio of progesterone receptor A (PR-A) to progesterone receptor B (PR-B) as an endpoint. It showed that nuclear factor kappa B p65-p50 heterodimer (NF-κB) increased the PR-A:PR-B ratio, and that higher doses of NF-κB shortened the time to reach the PR-A:PR-B ratio observed in labour. The model assumes that NF-κB is a marker of infection so these results reflect the known association between infection and preterm birth. This is an encouraging and useful first step towards modelling preterm labour, however the model does not include the molecular interactions upstream of NF-κB activation that initiate the whole pathway, and so does not allow *in silico* exploration of the importance of these interactions to the system. Additionally, the model does not include the complex interactions between molecules at an intracellular level and therefore risks oversimplifying the system.

Here, we use published data to build a comprehensive model of the intracellular signalling pathway that activates NF-κB p65-p50 in response to lipopolysaccharide (LPS). This is an important candidate signalling pathway in infection-induced preterm labour. The actions of LPS and NF-κB are well characterised: LPS is a gram negative bacterial endotoxin that triggers an inflammatory response in many cells including uterine smooth muscle cells [9]. LPS is often used in animal and culture studies to mimic intrauterine infection which subsequently induces preterm labour [10]–[13], therefore the actions of LPS could be considered to replicate the actions of the initiator of some cases of infection-induced preterm labour. NF-κB is a protein complex transcription factor with a particular role in the immune response to infection. It is activated in response to pro-inflammatory stimuli [14], but also regulates the transcription of inflammatory genes [15]–[17]. NF-κB activity increases in human labour, particularly in the fetal membranes [18], but also in the myometrium where labour is associated with an increase in the NF-κB p65-p50 heterodimer in pregnancy and labouring tissue compared to non-labouring tissue [19]. In this way NF-κB may act as a feed-forward mechanism for the inflammatory events associated with labour [20]. Therefore, in a uterine smooth muscle cell this signalling pathway is likely to be involved in triggering preterm labour in response to intrauterine infection. The pathway has been modelled previously in scenarios outwith pregnancy [21]–[23], but we are the first not only to model in pregnancy but also to include the molecular interactions upstream of IKK (IκB kinase), which include events from LPS to IKK and the production and action of TNFα (tumour necrosis factor alpha). Therefore our model allows us to assess the importance of these upstream interactions to the behaviour of the system. To our knowledge, this is the first attempt to generate a kinetic model specifically to improve understanding of parturition. The model is based on ordinary differential equations (ODEs), which have a well-established biophysical basis and straightforward molecular interpretation, and are therefore the most widely-used method to model signalling pathways [24]. ODE models are very comprehensive because they include all the known molecular entities in a system and quantitatively describe the kinetics of each physical interaction between them. This increases the likelihood that the model will allow us to fully understand and manipulate the complex behaviour of the system [24].

## Methods

To provide information on the LPS-NF-κB p65-p50 pathway structure and reaction kinetics we extensively searched the abstract repository PubMed (http://www.ncbi.nlm.nih.gov/pubmed/) and the pathway information resources KEGG (http://www.genome.jp/kegg/), Nature Pathway Interaction Database (http://pid.nci.nih.gov/) and Reactome (http://www.reactome.org/). Previously described computational models of NF-κB activation in scenarios outwith pregnancy [21]–[23] were also useful for finding reaction kinetics and were accessed via Biomodels (http://www.ebi.ac.uk/biomodels-main/), the online database of peer-reviewed published models.

We built a formalised pathway diagram using modified Edinburgh Pathway Notation in the graph editing application yED (yWorks, Germany).

We chose to build a deterministic-continuous (concentrations of entities change over time as a result of rate reactions, with no random variables introduced) model based on ordinary differential equations (ODEs) because this is the most common approach to modelling signalling pathways [24]. Kinetic models are highly detailed and require specific information about initial concentrations of reactants and rates of reactions (kinetic parameters). Therefore attempting to build a kinetic ODE model using solely published data is a challenge and a good test of the current level of knowledge and data accessibility. We considered using a partial differential equation (PDE) approach, which often allows more indepth kinetic analysis. However, the PDE approach requires even more specific data on spatial distribution of molecules within cells. Therefore, due to limited data availability, we favoured an ODE approach.

Here we have modelled the LPS-NF- κB p65-p50 pathway according to the suggestions put forward by Covert *et al*. [22] who built a computational model to study LPS-induced NF-κB activity in mouse embryo fibroblasts (MEFs). The pathway is described in detail in Text S1.

We developed our model using the modelling tool Copasi [25]. The structure of the IκB-NF-κB signalling portion of the model is largely based on a high quality model developed by Hoffmann *et al.* [21]. The final model was developed through successive rounds of model building and simulation within Copasi. After updating the model to steady-state concentrations with LPS = 0 µmol/ml, time course simulations were run with LPS arbitratily set at 1 µmol/ml.

## Results

### The Literature Search

**Figure 1:**
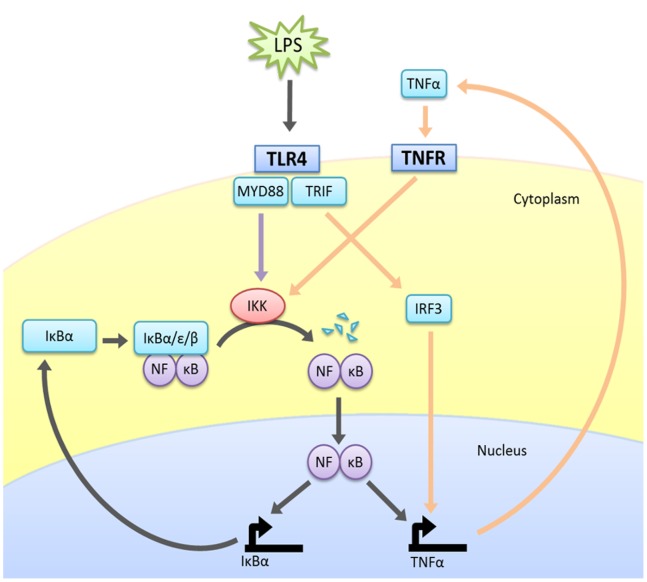
A simplified overview of the LPS-induced NF-κB signalling pathway. IκB, nuclear factor of kappa light polypeptide gene enhancer in B-cells inhibitor (alpha, beta and epsilon isoforms are incorporated into the model); IKK, IκB kinase; LPS, lipopolysaccharide; MyD88, myeloid differentiation primary response gene 88; NF-κB, nuclear factor kappa B; TLR4, toll-like receptor 4; TNFα, tumour necrosis factor alpha; TNFR, tumour necrosis factor receptor; TRIF, Tir-Domain-Containing Adapter-Inducing Interferon-β.

Figure 1 shows a simplified overview of the canonical LPS-NF-κB p65-p50 pathway, derived from generally accepted interactions in the literature. The pathway is described in more detail in Text S1. Much of this information was derived from cell types other than uterine smooth muscle cells. There was insufficient data in the literature to confirm whether any deviations of this standard pathway occur in uterine smooth muscle cells and in the scenario of pregnancy.

### Graphical Depiction

We took the individual molecular interactions, which are well characterised in the experimental literature, and built them into a single detailed graphical depiction of the canonical pathway (figure S1). This diagram is built using the standardised graphical notation Modified Edinburgh Pathway Notation (mEPN) [26]. It depicts every entity used in the final model, the reactions they are involved in, and in what way they react (binding, phosphorylation, etc.). This graphical depiction acts as a blueprint to the static structure of the model. To our knowledge, this is the first standardised graphical depiction of this pathway.

### Kinetic Model Structure and Parameters

A kinetic model was built using known parameters for each of the processes shown in the graphical depiction. The model reaction and kinetic equations used in the final model are listed in the supplementary material (Table S1). Table S2 in the supplementary material lists the initial concentrations of molecular species used in the model. The full model, encoded in SBML format, is available in the Biomodels database (http://www.ebi.ac.uk/biomodels-main/MODEL1303230000) and the supplementary material of this article.

An extensive search of the literature retrieved no data on time course behaviour or kinetic parameter values specific to uterine smooth muscle cells, so for the IKK-NF-κB portion of the model, kinetic values were taken from Hoffmann et al.’s model derived from experiments on mouse embryo fibroblasts (MEFs) [21]. Kinetic values for the novel reactions we included upstream of NF-κB activation, for example LPS to IKK, and production and actions of TNFα) were not available in the literature and were therefore imputed to fit the time course NF-κB activity profile observed by Covert et al. [22] in LPS-treated MEFs. Data-fitting is a standard technique in computational modelling and can be achieved automatically through algorithms that find optimal values [24], however in this case, we adjusted parameters manually because data was sparse.

### Steady State Behaviour

**Figure 2:**
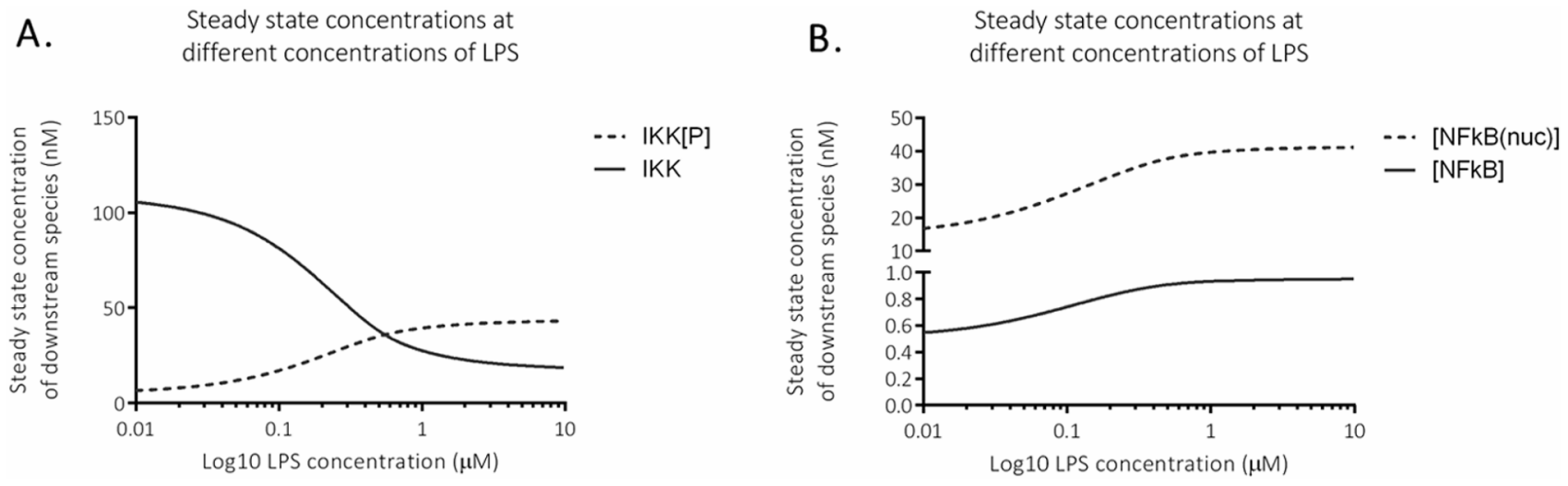
Steady state concentrations at different doses of LPS. A. *In silico* simulation of steady state concentrations of inactive and phosphorylated IKK, and B. *In silico* simulation of steady state concentrations of free nuclear and cytoplasmic NF-κB.

We ran the model to steady state (i.e. there is no further change in concentrations over time) using different concentrations of LPS and found that both IKK (active and inactive) and free NF-κB (nuclear and cytoplasmic) show a dose response (Figure 2). At LPS doses over 0.4, there is a switch from the majority of IKK being inactive to the majority being phosphorylated at steady state. Concentrations of nuclear and cytoplasmic NF-κB both increase with higher doses of LPS because LPS treatment leads to an increase in free cytoplasmic NF-κB via phosphorylated IKK, and this free cytoplasmic NF-κB then translocates to the nucleus.

### Time Course Behaviour

**Figure 3:**
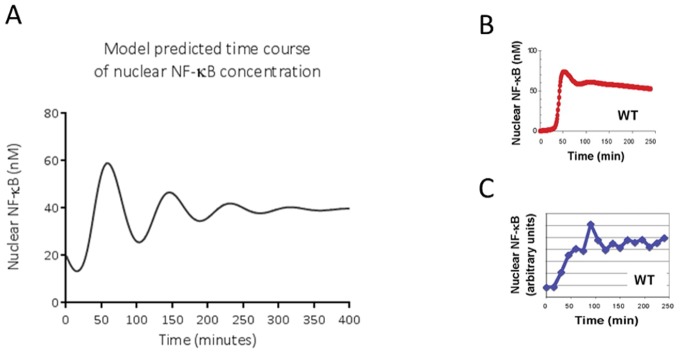
NF-κB time course behaviour of A. Concentrations of nuclear NF-κB over time, simulated using the *in silico* model described here, B. Concentrations of nuclear NF-κB over time, simulated using the *in silico* model described by Covert et al., C. Experimental data from LPS-treated mouse embryo fibroblasts as described by Covert et al. [22]. C is reprinted from Covert, M. W., Leung, T. H., Gaston, J. E., & Baltimore, D. (2005). Achieving stability of lipopolysaccharide-induced NF-kappaB activation. *Science (New York, N.Y.)*, *309*(5742), 1854-7. under a CC BY license, with permission from AAAS, original copyright 2005.

**Figure 4:**
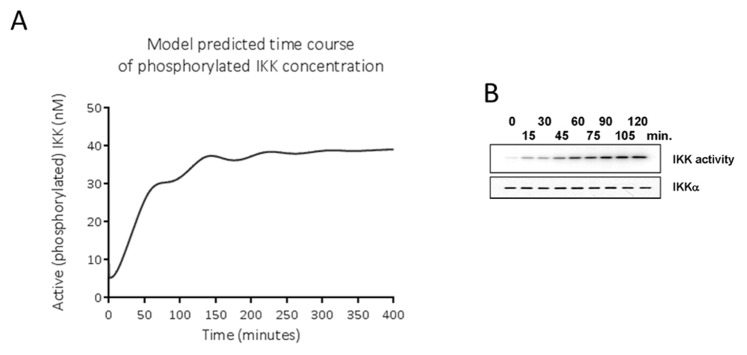
IKK time course behaviour. A. Concentrations of nuclear IKK over time, simulated using the *in silico* model described here, B. Experimental data from LPS-treated mouse embryo fibroblasts as described by Covert et al. [22]. B is reprinted from Covert, M. W., Leung, T. H., Gaston, J. E., & Baltimore, D. (2005). Achieving stability of lipopolysaccharide-induced NF-kappaB activation. *Science (New York, N.Y.)*, *309*(5742), 1854-7. under a CC BY license, with permission from AAAS, original copyright 2005.

The model mimics *in silico* the activation of NF-κB p65-p50 over time that Covert et al. described *in vitro* in time course experiments on LPS-treated wild-type MEFs, and *in silico* in their model of IKK-NF-κB signalling (Figure 3). Nuclear (active) concentration of NF-κB shows damped oscillatory behaviour. The model also mimics *in silico* the time-course concentration of phosphorylated IKK found by Covert et al. *in vitro* (Figure 4). Our *in silico* model mimics the pattern of this behaviour, although not to the exact degree; the timing, number of oscillations and exact concentrations are not the same as found by Covert et al. *in vitro*.

The model predicts that the concentration of the phosphorylated (active) form of IKK should increase with a small amount of oscillation and reach a maximum around 4 hours after LPS treatment. Covert et al.’s Western blot analysis suggests a similar pattern, but with a faster, steadier increase in phosphorylated IKK, reaching a maximum at around two hours after treatment.

**Figure 5:**
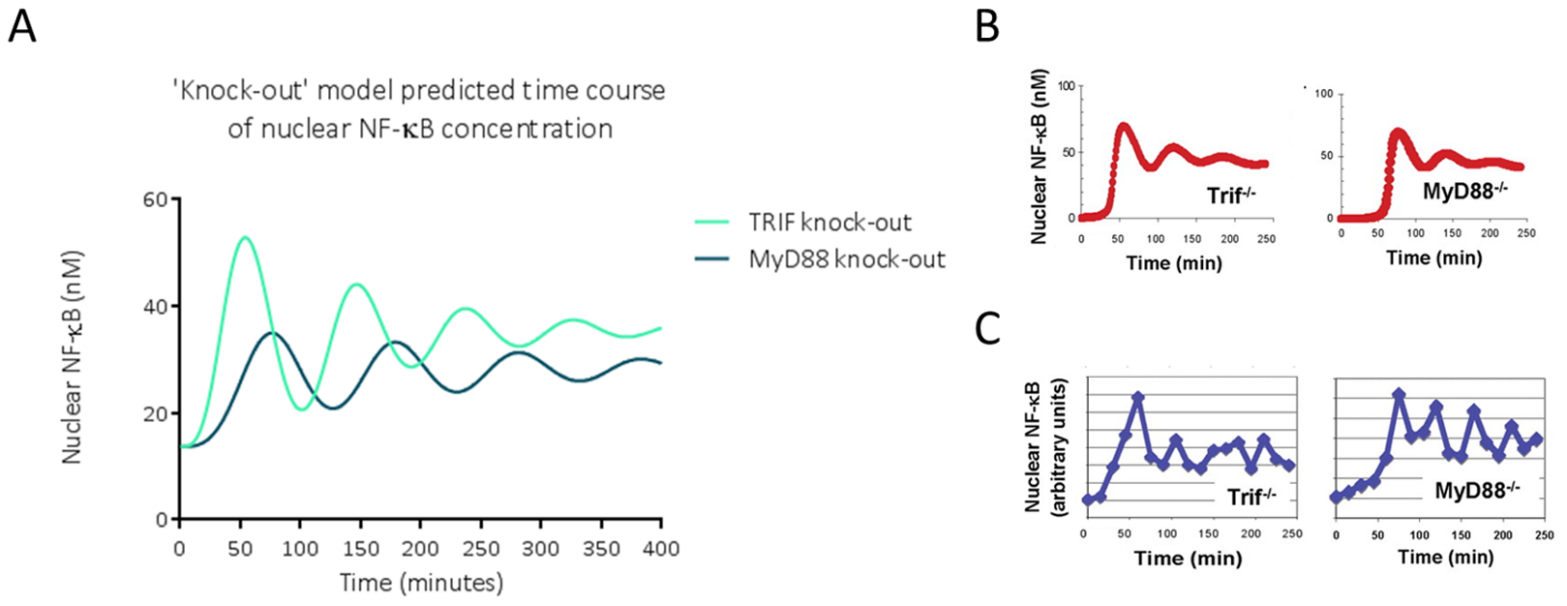
NF-κB time course behaviour in TRIF and MyD88 knock-out conditions. A. Concentrations of nuclear NF-κB over time, simulated using TRIF and MyD88 knock-out versions of the *in silico* model described here, B. Concentrations of nuclear NF-κB over time, simulated using TRIF and MyD88 knock-out versions of the *in silico* model described by Covert et al., C. Experimental *in vitro* data from LPS-treated TRIF or MyD88 knock-out mouse embryo fibroblasts as described by Covert et al. [22]. B and C are reprinted from Covert, M. W., Leung, T. H., Gaston, J. E., & Baltimore, D. (2005). Achieving stability of lipopolysaccharide-induced NF-kappaB activation. *Science (New York, N.Y.)*, *309*(5742), 1854-7. under a CC BY license, with permission from AAAS, original copyright 2005.

The model mimics *in silico* the activation of NF-κB over time that Covert et al. found in TRIF (Tir-Domain-Containing Adapter-Inducing Interferon-β) and MyD88 (Myeloid Differentiation Primary Response Gene 88) (Figure 5) knock-out cells *in vitro*. After LPS treatment of both knock-out cells, Covert et al.’s time-course experiments showed increased oscillatory NF-κB activation compared to wild-type cells, and the initiation of NF-κB activation was delayed by around 30 minutes in MyD88 knock-out compared to wild-type cells. The authors argued that this 30 minute delay occurs because the TRIF-dependent pathway relies on the synthesis and actions of TNFα. Whereas Covert et al. could only mimic this behaviour *in silico* by introducing an artificial delay to mimic MyD88 knock-out, our extended model allows us to ‘knock-out’ MyD88 or TRIF directly, by fixing their concentrations at 0 before running steady state and time course simulations as described above. Our model also includes the reactions involved in the synthesis of TNFα and its autocrine actions on the cell. Therefore we are able to test the downstream effects of MyD88 and TRIF knock-out more naturally. Our model successfully predicts increased oscillations in NF-κB activation when TRIF or MyD88 is ‘knocked out’, and there is a simulated delay in NF-κB activation when MyD88 is ‘knocked out’. Again, although the model captures the general pattern of the *in vitro* data, it is not quantitatively accurate.

## Discussion

To our knowledge, this is the first kinetic model of a signalling pathway relevant to infection-induced preterm labour. Using only published data, we have produced a graphical depiction and kinetic model of LPS-induced NF-κB p65-p50 activation. Previous models of NF-κB activity published in scenarios outwith pregnancy use IKK as an input to allow the model to be adapted to simulate NF-κB activity following any treatment [21]–[23]. However, in a pregnancy scenario, LPS is a more appropriate input than IKK because LPS could be considered the initiator of some cases of infection-induced preterm labour. Therefore, we extended previous models by using LPS as an input and explicitly modelling molecular interactions upstream of IKK activation, including LPS to IKK and the production and action of TNFα. This allows closer analysis of the interactions that activate IKK and therefore affect downstream NF-kB activity. After validation using cells from human uterine smooth muscle cells, this will allow *in silico* testing of drugs targeting these upstream interactions.

Ours is the first attempt to explicitly model these upstream events appropriately. One previous attempt by Selvarajoo [27] was flawed because although the kinetic rate equations were based on mass action kinetics, they did not describe physical interactions between individual entities. For example, the first reaction in the Selvarajoo model, “TLR4<-> MyD88” (rate equation: Kf [TLR4] – Kr[MyD88]), describes a reaction where TLR4 is reversibly converted to MyD88, which does not represent the true physical interaction between these two molecules.

Explicitly modelling the upstream events produces a more complete model that is able to reproduce *in silico* the published behaviour of the system *in vitro* in wild-type, MyD88 knock-out and TRIF knock-out MEFs. Although our model can simulate the pattern of the *in vitro* behaviour, the exact timing, number of oscillations and exact concentrations were different. However, this is unlikely to invalidate the model because the kinetics of the pathway are also likely to alter in different experimental conditions and in different cell types. There are undoubtedly more LPS targets that could be incorporated into extended versions of the model to make it more comprehensive and improve its potential to make predictions about the relative importance of different parts of the pathway.

We found no published data on the structure or kinetics of the LPS-NF-κB pathway in uterine smooth muscle cells and therefore cannot confirm that the pathway deviates from that described in MEFs. Although this is a major limitation of our model, we do not anticipate that there would be any major deviations because the pathway appears to be well conserved [28]. However, wet lab experiments using uterine smooth muscle cells should be conducted to validate the model in this cell type. The lack of available published data also highlights the need for the publication of detailed data from time course experiments to aid with model building.

We have provided proof of concept that it is possible to build computational models of signalling pathways relevant to labour. When validated using wet lab experiments on cells derived from human gestational tissue (for example, uterine smooth muscle cells), such models could be used for drug testing *in silico*, providing a rapid, safe, economical and ethical strategy to identify candidate effective therapies for further testing. Thus, these models have the potential to improve our understanding of parturition and translate into improved pregnancy outcomes.

## Supporting Information

Figure S1 **Pathway depiction created using modified Edinburgh Pathway Notation (mEPN).** A key to this graphical notation is provided at http://www.mepn-pathway.org/. A full description of the pathway is provided in Text S1.
(PDF)

Table S1 **Summary of reactions and reaction parameters used in the model.** *Red italics* = parameters derived during model fitting to experimental data from Covert et al. [22]; *all other values* = parameters used in Hoffmann et al.’s model of NF-κB signalling [21]. *v* = reaction rate, *kf* = rate of the forward reaction, *kr* = rate of the reverse reaction.
(PDF)

Table S2 **Initial (pre-steady state) concentrations of species used in the model.** All values were set to 1 µmol/ml if they were assumed to be present at time = 0 s and 0 µM if they were assumed to be absent. NF-κB was set to 0.1 µmol/ml as in Hoffmann et al. [21]. Concentrations marked with an asterisk were ‘fixed’ at their initial concentrations to avoid overcomplicating the model by modelling synthesis and degradation of these species.
(PDF)

Text S1 **LPS-induced NF-kappa B activation as described in the literature.**
(DOCX)

Model File S1 **The kinetic model encoded in SBML format.**
(XML)
