# Supplementary figures and images for: A Computational Model of Lipopolysaccharide-Induced Nuclear Factor Kappa B Activation: A Key Signalling Pathway in Infection-Induced Preterm Labour
Source: PLoS One. 2013 Jul 30;8(7):e70180. doi: 10.1371/journal.pone.0070180 (PMC3736540; doi:10.1371/journal.pone.0070180)

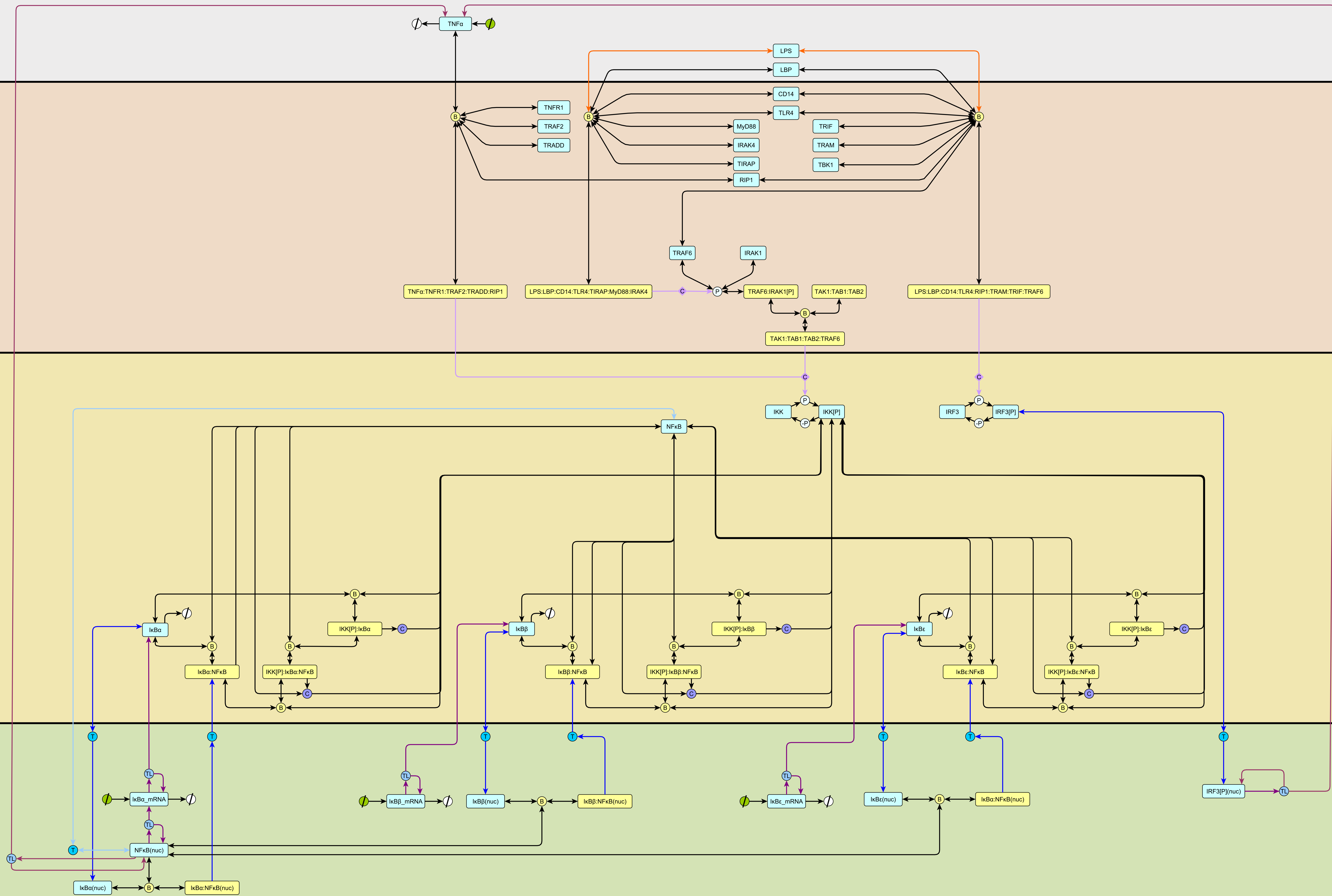

Supplement: Figure S1 — Pathway depiction created using modified Edinburgh Pathway Notation (mEPN). A key to this graphical notation is provided at http://www.mepn-pathway.org/. A full description of the pathway is provided in Text S1. (PDF) [file pone.0070180.s001.pdf]
