# Supplementary material for: A Computational Model of Lipopolysaccharide-Induced Nuclear Factor Kappa B Activation: A Key Signalling Pathway in Infection-Induced Preterm Labour
Source: PLoS One. 2013 Jul 30;8(7):e70180. doi: 10.1371/journal.pone.0070180 (PMC3736540; doi:10.1371/journal.pone.0070180)
Supplement: Table S1 — Summary of reactions and reaction parameters used in the model. Red italics = parameters derived during model fitting to experimental data from Covert et al. [22]; all other values = parameters used in Hoffmann et al.’s model of NF-κB signalling [21]. v = reaction rate, kf = rate of the forward reaction, kr = rate of the reverse reaction. (PDF) [file pone.0070180.s002.pdf]

	Reaction name	Reaction equation	Rate equation	Kinetic parameters	
1	MYD881) Receptor Complex Formation	LPS + LBP + CD14 + TLR4 + MyD88 + IRAK4 + TIRAP = LPS:LBP:CD14:TLR4:TIRAP:MyD88:IRAK4	$v^{1} = kf^{1} \cdot [\text{LPS}] \cdot [\text{LBP}] \cdot [\text{CD14}] \cdot [\text{TLR4}] \cdot [\text{MYD88}] \cdot [\text{IRAK4}] \cdot [\text{TIRAP}] - kr^{1} \cdot [\text{LPS:LBP:CD14:TLR4:TIRAP:MyD88:IRAK4}]$	kf^{1}<br>kr^{1}	0.0001 ml^6/(μmol^6*s)<br>0.0001 1/s
2	MYD882) IRAK1 and TRAF6 Phosphorylation	IRAK1 + TRAF6 = TRAF6:IRAK1[P]; LPS:LBP:CD14:TLR4:TIRAP:MyD88:IRAK4	$v^{2} = kf^{2} \cdot [\text{LPS:LBP:CD14:TLR4:TIRAP:MyD88:IRAK4}] \cdot [\text{TRAF6}] \cdot [\text{IRAK1}] - kr^{2} \cdot [\text{TRAF6:IRAK1[P]}]$	kf^{2}<br>kr^{2}	0.001 ml^2/(μmol^2*s)<br>0.001 1/s
3	MYD883) TAK/TAB Binds to TRAF6	TRAF6:IRAK1[P] + TAK1:TAB1:TAB2 = TAK1:TAB1:TAB2:TRAF6	$v^{3} = kf^{3} \cdot [\text{TRAF6:IRAK1[P]}] \cdot [\text{TAK1:TAB1:TAB2}] - kr^{3} \cdot [\text{TAK1:TAB1:TAB2:TRAF6}]$	kf^{3}<br>kr^{3}	0.003 ml/(μmol*s)<br>0.01 1/s
4	MYD884) IKK Phosphorylation by TAK1	IKK -> IKK[P]; TAK1:TAB1:TAB2:TRAF6	$v^{4} = \frac{k^{4} \cdot [\text{TAK1:TAB1:TAB2:TRAF6}] \cdot [\text{IKK}]}{km^{4} + [\text{IKK}]}$	k^{4}<br>km^{4}	0.1 1/s<br>0.1 μmol/ml
5	MYD885) IKK[P] Dephosphorylation	IKK[P] -> IKK	$v^{5} = kf^{5} \cdot [\text{IKK[P]}]$	kf^{5}	0.1 1/s
6	TRIF01) Receptor Complex Formation	LPS + LBP + CD14 + TLR4 + TRIF + TRAM + RIP1 + TBK1/IKKe = LPS:LBP:CD14:TLR4:RIP1:TRAM:TRIF:TBK1/IKKe	$v^{6} = kf^{6} \cdot [\text{LPS}] \cdot [\text{LBP}] \cdot [\text{CD14}] \cdot [\text{TLR4}] \cdot [\text{TRIF}] \cdot [\text{TRAM}] \cdot [\text{RIP1}] \cdot [\text{TBK1/IKKe}] - kr^{6} \cdot [\text{LPS:LBP:CD14:TLR4:TRAM:TRIF:TBK1/IKKe}]$	kf^{6}<br>kr^{6}	0.0001 ml^7/(μmol^7*s)<br>0.0001 1/s
7	TRIF02) IRF3 Phosphorylation	IRF3 -> IRF3[P]; LPS:LBP:CD14:TLR4:RIP1:TRAM:TRIF:TBK/IKKe	$v^{7} = \frac{k^{7} \cdot [\text{LPS:LBP:CD14:TLR4:RIP1:TRAM:TRIF:TBK/IKKe}] \cdot [\text{IRF3}]}{km^{7} + [\text{IRF3}]}$	k^{7}<br>km^{7}	0.1 1/s<br>0.1 μmol/ml
8	TRIF03) IRF3 Dephosphorylation	IRF3[P] -> IRF3	$v^{8} = kf^{8} \cdot [\text{IRF3[P]}]$	kf^{8}	0.1 1/s
9	TRIF04) IRF3 Nuclear Import/Export	IRF3[P] = IRF3[P](nuc)	$v^{9} = kf^{9} \cdot [\text{IRF3[P]}] - kr^{9} \cdot [\text{IRF3[P](nuc)}]$	kf^{9}<br>kr^{9}	0.1 1/s<br>0.1 1/s
10	TRIF05) Inducible TNFa Synthesis	2 * IRF3[P](nuc) -> TNFa + 2 * IRF3[P](nuc)	$v^{10} = kf^{10} \cdot 2 \cdot [\text{IRF3[P](nuc)}]$	kf^{10}	0.02 ml/(μmol*s)
11	TRIF06) Constitutive TNFa Synthesis	source -> TNFa	$v^{11} = kf^{11} \cdot [\text{source}]$	kf^{11}	0.001 1/s
12	TRIF07)Inducible TNFa Synthesis by NFkB	2 * NFkB(nuc) -> TNFa + 2 * NFkB(nuc)	$v^{12} = kf^{12} \cdot 2 \cdot [\text{NFkB(nuc)}]$	kf^{12}	0.001 ml/(μmol*s)
13	TRIF08) TNFa Degradation	TNFa -> sink	$v^{13} = kf^{13} \cdot [\text{TNFa}]$	kf^{13}	0.1 1/s
14	TRIF09) TNFa Receptor Complex Formation	TNFa + TNFR1 + TRAF2 + TRADD + RIP1 = TNFa:TNFR1:TRAF2:TRADD:RIP1	$v^{14} = kf^{14} \cdot [\text{TNFa}] \cdot [\text{TNFR1}] \cdot [\text{TRAF2}] \cdot [\text{TRADD}] \cdot [\text{RIP1}] - kr^{14} \cdot [\text{TNFa:TNFR1:TRAF:2:TRADD:RIP1}]$	kf^{14}<br>kr^{14}	0.1 ml^4/(μmol^4*s)<br>0.1 1/s
15	TRIF10) IKK Phosphorylation by RIP1	IKK -> IKK[P]; TNFa:TNFR1:TRAF2:TRADD:RIP1	$v^{15} = \frac{k^{15} \cdot [\text{TNFa:TNFR1:TRAF2:TRADD:RIP1}] \cdot [\text{IKK}]}{km^{15} + [\text{IKK}]}$	k^{15}<br>km^{15}	0.1 1/s<br>0.1 μmol/ml
16	NFkB01) IkBa:NFkB Binding	IkBa + NFkB = IkBa:NFkB	$v^{16} = kf^{16} \cdot [\text{IkBa}] \cdot [\text{NFkB}] - kr^{16} \cdot [\text{IkBa:NFkB}]$	kf^{16}<br>kr^{16}	0.5 ml/(μmol*s)<br>0.0005 1/s
17	NFkB02) IKK:IkBa:NFkB Binding (1)	IKK[P] + IkBa:NFkB = IKK[P]:IkBa:NFkB	$v^{17} = kf^{17} \cdot [\text{IKK[P]}] \cdot [\text{IkBa:NFkB}] - kr^{16} \cdot [\text{IKK[P]:IkBa:NFkB}]$	kf^{17}<br>kr^{17}	0.185 ml/(μmol*s)<br>0.0125 1/s
18	NFkB03) IkBb:NFkB Binding	IkBb + NFkB = IkBb:NFkB	$v^{18} = kf^{18} \cdot [\text{IkBb}] \cdot [\text{NFkB}] - kr^{18} \cdot [\text{IkBb:NFkB}]$	kf^{18}<br>kr^{18}	0.5 ml/(μmol*s)<br>0.0005 1/s
19	NFkB04) IKK:IkBb:NFkB Binding (1)	IKK[P] + IkBb:NFkB = IKK[P]:IkBb:NFkB	$v^{19} = kf^{19} \cdot [\text{IKK[P]}] \cdot [\text{IkBb:NFkB}] - kr^{19} \cdot [\text{IKK[P]:IkBb:NFkB}]$	kf^{19}<br>kr^{19}	0.048 ml/(μmol*s)<br>0.00175 1/s
20	NFkB05) IkBe:NFkB Binding	IkBe + NFkB = IkBe:NFkB	$v^{20} = kf^{20} \cdot [\text{IkBe}] \cdot [\text{NFkB}] - kr^{20} \cdot [\text{IkBe:NFkB}]$	kf^{20}<br>kr^{20}	0.5 ml/(μmol*s)<br>0.0005 1/s
21	NFkB06) IKK:IkBe:NFkB Binding (1)	IKK[P] + IkBe:NFkB = IKK[P]:IkBe:NFkB	$v^{21} = kf^{21} \cdot [\text{IKK[P]}] \cdot [\text{IkBe:NFkB}] - kr^{21} \cdot [\text{IKK[P]:IkBe:NFkB}]$	kf^{21}<br>kr^{21}	0.07 ml/(μmol*s)<br>0.00175 1/s
22	NFkB07) IKK:IkBa:NFkB Catalysis	IKK[P]:IkBa:NFkB -> IKK[P] + NFkB	$v^{22} = kf^{22} \cdot [\text{IKK[P]:IkBa:NFkB}]$	kf^{22}	0.0204 1/s
23	NFkB08) IKK:IkBb:NFkB Catalysis	IKK[P]:IkBb:NFkB -> IKK[P] + NFkB	$v^{23} = kf^{23} \cdot [\text{IKK[P]:IkBb:NFkB}]$	kf^{23}	0.0075 1/s
24	NFkB09) IKK:IkBe:NFkB Catalysis	IKK[P]:IkBe:NFkB -> IKK[P] + NFkB	$v^{24} = kf^{24} \cdot [\text{IKK[P]:IkBe:NFkB}]$	kf^{24}	0.011 1/s
25	NFkB10) IkBa:NFkB Constitutive Degradation	IkBa:NFkB -> NFkB	$v^{25} = kf^{25} \cdot [\text{IkBa:NFkB}]$	kf^{25}	2.25E-05 1/s
26	NFkB11) IkBb:NFkB Constitutive Degradation	IkBb:NFkB -> NFkB	$v^{26} = kf^{26} \cdot [\text{IkBb:NFkB}]$	kf^{26}	2.25E-05 1/s
27	NFkB12) IkBe:NFkB Constitutive Degradation	IkBe:NFkB -> NFkB	$v^{27} = kf^{27} \cdot [\text{IkBe:NFkB}]$	kf^{27}	2.25E-05 1/s
28	NFkB13) NFkB Nuclear Import/Export	NFkB = NFkB(nuc)	$v^{28} = kf^{28} \cdot [\text{NFkB}] - kr^{28} \cdot [\text{NFkB(nuc)}]$	kf^{28}<br>kr^{28}	0.09 1/s<br>8.00E-05 1/s
29	NFkB14) Nuclear IkBa:NFkB Binding	IkBa(nuc) + NFkB(nuc) = IkBa:NFkB(nuc)	$v^{29} = kf^{29} \cdot [\text{IkBa(nuc)}] \cdot [\text{NFkB(nuc)}] - kr^{29} \cdot [\text{IkBa:NFkB(nuc)}]$	kf^{29}<br>kr^{29}	0.5 ml/(μmol*s)<br>0.0005 1/s
30	NFkB15) Nuclear IkBb:NFkB Binding	IkBb(nuc) + NFkB(nuc) = IkBb:NFkB(nuc)	$v^{30} = kf^{30} \cdot [\text{IkBb(nuc)}] \cdot [\text{NFkB(nuc)}] - kr^{30} \cdot [\text{IkBb:NFkB(nuc)}]$	kf^{30}<br>kr^{30}	0.5 ml/(μmol*s)<br>0.0005 1/s
31	NFkB16) Nuclear IkBe:NFkB Binding	IkBe(nuc) + NFkB(nuc) = IkBe:NFkB(nuc)	$v^{31} = kf^{31} \cdot [\text{IkBe(nuc)}] \cdot [\text{NFkB(nuc)}] - kr^{31} \cdot [\text{IkBe:NFkB(nuc)}]$	kf^{31}<br>kr^{31}	0.5 ml/(μmol*s)<br>0.0005 1/s
32	NFkB17) Constitutive IkBa mRNA Synthesis	source -> IkBa_mRNA	$v^{32} = kf^{32} \cdot [\text{source}]$	kf^{32}	1.54E-06 1/s
33	NFkB18) Inducible IkBa mRNA Synthesis	2 * NFkB(nuc) -> IkBa_mRNA + 2 * NFkB(nuc)	$v^{33} = kf^{33} \cdot 2 \cdot [\text{NFkB(nuc)}]$	kf^{33}	0.0165 ml/(μmol*s)
34	NFkB19) IkBa mRNA degradation	IkBa_mRNA -> sink	$v^{34} = kf^{34} \cdot [\text{IkBa\_mRNA}]$	kf^{34}	0.00028 1/s
35	NFkB20) Constitutive IkBb mRNA Synthesis	source -> IkBb_mRNA	$v^{35} = kf^{35} \cdot [\text{source}]$	kf^{35}	1.78E-07 1/s
36	NFkB21) IkBb mRNA degradation	IkBb_mRNA -> sink	$v^{36} = kf^{36} \cdot [\text{IkBb\_mRNA}]$	kf^{36}	0.00028 1/s
37	NFkB22) Constitutive IkBe mRNA Synthesis	source -> IkBe_mRNA	$v^{37} = kf^{37} \cdot [\text{source}]$	kf^{37}	1.27E-07 1/s
38	NFkB23) IkBe mRNA degradation	IkBe_mRNA -> sink	$v^{38} = kf^{38} \cdot [\text{IkBe\_mRNA}]$	kf^{38}	0.00028 1/s
39	NFkB24) IKK:IkBa Binding	IKK[P] + IkBa = IKK[P]:IkBa	$v^{39} = kf^{39} \cdot [\text{IKK[P]}] \cdot [\text{IKBa}] - kr^{39} \cdot [\text{IKK[P]:IkBa}]$	kf^{39}<br>kr^{39}	0.0225 ml/(μmol*s)<br>0.00125 1/s
40	NFkB25) IkBa Translation	IkBa_mRNA -> IkBa + IkBa_mRNA	$v^{40} = kf^{40} \cdot [\text{IkBa\_mRNA}]$	kf^{40}	0.00408 1/s
41	NFkB26) IkBa Degradation	IkBa -> sink	$v^{41} = kf^{41} \cdot [\text{IkBa}]$	kf^{41}	0.000113 1/s
42	NFkB27) IkBa Nuclear Import/Export	IkBa = IkBa(nuc)	$v^{42} = kf^{42} \cdot [\text{IKBa}] - kr^{42} \cdot [\text{IkBa(nuc)}]$	kf^{42}<br>kr^{42}	0.0003 1/s<br>0.0002 1/s
43	NFkB28) IKK:IkBb Binding	IKK[P] + IkBb = IKK[P]:IkBb	$v^{43} = kf^{43} \cdot [\text{IKK[P]}] \cdot [\text{IKBb}] - kr^{43} \cdot [\text{IKK[P]:IkBb}]$	kf^{43}<br>kr^{43}	0.006 ml/(μmol*s)<br>0.00175 1/s
44	NFkB29) IkBb Translation	IkBb_mRNA -> IkBb + IkBb_mRNA	$v^{44} = kf^{44} \cdot [\text{IkBb\_mRNA}]$	kf^{44}	0.00408 1/s
45	NFkB30) IkBb Degradation	IkBb -> sink	$v^{45} = kf^{45} \cdot [\text{IkBb}]$	kf^{45}	0.000113 1/s
46	NFkB31) IkBb Nuclear Import/Export	IkBb = IkBb(nuc)	$v^{46} = kf^{46} \cdot [\text{IKBb}] - kr^{46} \cdot [\text{IkBb(nuc)}]$	kf^{46}<br>kr^{46}	0.00015 1/s<br>0.0001 1/s
47	NFkB32) IKK:IkBe Binding	IKK[P] + IkBe = IKK[P]:IkBe	$v^{47} = kf^{47} \cdot [\text{IKK[P]}] \cdot [\text{IKBe}] - kr^{47} \cdot [\text{IKK[P]:IkBe}]$	kf^{47}<br>kr^{47}	0.009 ml/(μmol*s)<br>0.00175 1/s
48	NFkB33) IkBe Translation	IkBe_mRNA -> IkBe + IkBe_mRNA	$v^{48} = kf^{48} \cdot [\text{IkBe\_mRNA}]$	kf^{48}	0.00408 1/s
49	NFkB34) IkBe Degradation	IkBe -> sink	$v^{49} = kf^{49} \cdot [\text{IkBe}]$	kf^{49}	0.000113 1/s
50	NFkB35) IkBe Nuclear Import/Export	IkBe = IkBe(nuc)	$v^{50} = kf^{50} \cdot [\text{IKBe}] - kr^{50} \cdot [\text{IkBe(nuc)}]$	kf^{50}<br>kr^{50}	0.00015 1/s<br>0.0001 1/s
51	NFkB36) IKK:IkBa:NFkB Binding (2)	IKK[P]:IkBa + NFkB = IKK[P]:IkBa:NFkB	$v^{51} = kf^{51} \cdot [\text{IKK[P]:IkBa}] \cdot [\text{NFkB}] - kr^{51} \cdot [\text{IKK[P]:IkBa:NFkB}]$	kf^{51}<br>kr^{51}	0.5 ml/(μmol*s)<br>0.0005 1/s
52	NFkB37) IkBa:NFkB Nuclear Export	IkBa:NFkB(nuc) -> IkBa:NFkB	$v^{52} = kf^{52} \cdot [\text{IkBa:NFkB(nuc)}]$	kf^{52}	0.0138 1/s
53	NFkB38) IKK:IkBb:NFkB Binding (2)	IKK[P]:IkBb + NFkB = IKK[P]:IkBb:NFkB	$v^{53} = kf^{53} \cdot [\text{IKK[P]:IkBb}] \cdot [\text{NFkB}] - kr^{53} \cdot [\text{IKK[P]:IkBb:NFkB}]$	kf^{53}<br>kr^{53}	0.5 ml/(μmol*s)<br>0.0005 1/s
54	NFkB39) IkBb:NFkB Nuclear Export	IkBb:NFkB(nuc) -> IkBb:NFkB	$v^{54} = kf^{54} \cdot [\text{IkBb:NFkB(nuc)}]$	kf^{54}	0.0052 1/s
55	NFkB40) IKK:IkBe:NFkB Binding (2)	IKK[P]:IkBe + NFkB = IKK[P]:IkBe:NFkB	$v^{55} = kf^{55} \cdot [\text{IKK[P]:IkBe}] \cdot [\text{NFkB}] - kr^{55} \cdot [\text{IKK[P]:IkBe:NFkB}]$	kf^{55}<br>kr^{55}	0.5 ml/(μmol*s)<br>0.0005 1/s
56	NFkB41) IkBe:NFkB Nuclear Export	IkBe:NFkB(nuc) -> IkBe:NFkB	$v^{56} = kf^{56} \cdot [\text{IkBe:NFkB(nuc)}]$	kf^{56}	0.0052 1/s
57	NFkB42) IKK:IkBa Catalysis	IKK[P]:IkBa -> IKK[P]	$v^{57} = kf^{57} \cdot [\text{IKK[P]:IkBa}]$	kf^{57}	0.00407 1/s
58	NFkB43) IKK:IkBb Catalysis	IKK[P]:IkBb -> IKK[P]	$v^{58} = kf^{58} \cdot [\text{IKK[P]:IkBb}]$	kf^{58}	0.0015 1/s
59	NFkB44) IKK:IkBe Catalysis	IKK[P]:IkBe -> IKK[P]	$v^{59} = kf^{59} \cdot [\text{IKK[P]:IkBe}]$	kf^{59}	0.0022 1/s
