# Supplementary material for: A Computational Model of Lipopolysaccharide-Induced Nuclear Factor Kappa B Activation: A Key Signalling Pathway in Infection-Induced Preterm Labour
Source: PLoS One. 2013 Jul 30;8(7):e70180. doi: 10.1371/journal.pone.0070180 (PMC3736540; doi:10.1371/journal.pone.0070180)
Supplement: Table S2 — Initial (pre-steady state) concentrations of species used in the model. All values were set to 1 µmol/ml if they were assumed to be present at time = 0 s and 0 µM if they were assumed to be absent. NF-κB was set to 0.1 µmol/ml as in Hoffmann et al. [21]. Concentrations marked with an asterisk were ‘fixed’ at their initial concentrations to avoid overcomplicating the model by modelling synthesis and degradation of these species. (PDF) [file pone.0070180.s003.pdf]

Species	Initial concentration (μmol/ml)
CD14	1*
IkBa	0
IkBa(nuc)	0
IkBa:NFkB	0
IkBa:NFkB(nuc)	0
IkBa_mRNA	0
IkBb	0
IkBb(nuc)	0
IkBb:NFkB	0
IkBb:NFkB(nuc)	0
IkBb_mRNA	0
IkBe	0
IkBe(nuc)	0
IkBe:NFkB	0
IkBe:NFkB(nuc)	0
IkBe_mRNA	0
IKK	0.1
IKK[P]	0
IKK[P]:IkBa	0
IKK[P]:IkBa:NFkB	0
IKK[P]:IkBb	0
IKK[P]:IkBb:NFkB	0
IKK[P]:IkBe	0
IKK[P]:IkBe:NFkB	0
IRAK1	1
IRAK4	1*
IRF3	1
IRF3[P]	0
IRF3[P](nuc)	0
LBP	1*
LPS	1*
LPS:LBP:CD14:TLR4:RIP1:TRAM:TRIF:TBK/IKKe	0
LPS:LBP:CD14:TLR4:TIRAP:MyD88:IRAK4	0
MyD88	1*
NFkB	0.1
NFkB(nuc)	0
RIP1	1*
sink	0*
source	1*
TAK1:TAB1:TAB2	1*
TAK1:TAB1:TAB2:TRAF6	0
TBK1/IKKe	1*
TIRAP	1*
TLR4	1*
TNFa	0
TNFa:TNFR1:TRAF2:TRADD:RIP1	0
TNFR1	1*

TRADD	1*
TRAF2	1*
TRAF6	1*
TRAF6:IRAK1[P]	0
TRAM	1*
TRIF	1*
