# Supplementary material for: A Computational Model of Lipopolysaccharide-Induced Nuclear Factor Kappa B Activation: A Key Signalling Pathway in Infection-Induced Preterm Labour
Source: PLoS One. 2013 Jul 30;8(7):e70180. doi: 10.1371/journal.pone.0070180 (PMC3736540; doi:10.1371/journal.pone.0070180)
Supplement: Text S1 — LPS-induced NF-kappa B activation as described in the literature. (DOCX) [file pone.0070180.s004.docx]

# Supplementary information

**LPS-induced NF-kappa B activation as described in the literature.**

The pathway can be split into three modules: 1) The MyD88-dependent pathway, 2) The TRIF-dependent pathway, 3) IκB-NF-κB signalling.

The reactions that describe LPS activation of its receptor Toll Like Receptor 4 (TLR4) are common to both modules 1 and 2: LPS binds to LPS-binding protein (LBP)[1] and this complex binds to and activates CD14 on the cell membrane [2]. CD14 then helps transport the LPS-LBP complex to TLR4 where LPS activates the receptor [3–6].

Further downstream signalling occurs via two pathways: one dependent on Myeloid Differentiation Primary Response Gene 88 (MyD88) and the other dependent on Tir-Domain-Containing Adapter-Inducing Interferon-β (TRIF). The MyD88-dependent pathway is largely responsible for the expression of proinflammatory cytokines [7–12], and the main role of the TRIF-dependent pathway is to induce expression of co-stimulatory molecules and IFN-inducible genes [13–16]. NF-κB can be activated via either pathway, and *in vivo* the two pathways probably interact to maximise expression of inflammatory cytokines [17].

Module 1) The MyD88-dependent pathway

The MyD88-dependent pathway begins when TIRAP (toll-interleukin 1 receptor domain containing adaptor protein) is recruited to the cytoplasmic domain of TLR4 [18,19]. TIRAP recruits MyD88 [18–20], which binds IRAK4 (interleukin-1 receptor-associated kinase 4),[21,22] IRAK1 (interleukin-1 receptor-associated kinase 1)[23] and TRAF6 (TNF receptor associated factor 6) [24,25]. IRAK1 is phosphorylated by IRAK4 [21,26,27] and then the IRAK1/TRAF6 complex dissociates from the receptor[23,27]. TRAF6 then interacts with a complex consisting of the MAPK kinase kinase transforming growth factor-beta-activated kinase 1 (TAK1) and its binding proteins TAB1 and TAB2 [28]. The TRAF6/IRAK1/TAK1/TAB1/TAB2 complex is inactive until it translocates to the cytoplasm and forms a larger complex with the ubiquitin-conjugating enzymes Ubc13 and Uev1A. This ubiquitination activates TAK1 and marks IRAK1 for degradation by the proteasome [28,29]. TAK1 phosphorylates an IκB kinase (IKK) complex consisting of two catalytic subunits (IKKα and IKKβ) and a regulatory subunit (IKKγ) [30–33]. This phosphorylated IKK complex feeds into the IκB-NF-κB signalling module of the pathway.

Module 2) The TRIF-dependent pathway

Compared to the MyD88-dependent pathway, less is known about the TRIF-dependent module. Here we have modelled the pathway according to the suggestions put forward by Covert *et al*.[34]. They observed delayed NF-κB activation in MyD88-deficient mice embryo fibroblasts (MEFs) treated with LPS. They hypothesised the delay occurs because the TRIF-dependent pathway requires protein synthesis before NF-κB can be activated via a secondary signal in an autocrine manner. Microarray analysis of LPS stimulated MyD88-deficient MEFs showed significant upregulation of the tumour necrosis factor alpha (TNFα) transcript, which (along with the findings of additional validation experiments) led them to suggest that TNFα is the autocrine signal responsible for the late-phase activation of NF-κB in response to LPS.

The pathway they put forward begins when TRIF-related adapter molecule (TRAM) binds to active TLR4[35–37] and acts as a bridging adaptor connecting TLR4 to TRIF [36,38]. This complex activates two noncanonical IKKs, TANK-binding kinase 1(TBK1) and IKKε, [39–41] which phosphorylate Interferon Regulatory Factor 3 (IRF3) [39,40,42]. IRF3 then translocates to the nucleus and regulates transcription of TNFα. TNFα is secreted into the extracellular space where it binds to its membrane receptor TNFR1[43]. This leads to the recruitment of TRAF2, TRADD and RIP1. TRAF2 then recruits the IKK complex, which allows it to be phosphorylated by RIP1 [44–48].

Module 3) IκB-NF-κB signalling

In resting cells, NF-κB is sequestered in an inactive state in the cytoplasm by nuclear factor of kappa light polypeptide gene enhancer in B-cells inhibitor (IκB). Active IKK can bind to and phosphorylate IκB, which causes it to degrade and therefore release NF-κB [41,49]. Free NF-κB then translocates to the nucleus where it regulates transcription of target genes, including the IκB isoform IκBα [50–52]. The production of IκBα results in a negative feedback loop that causes oscillations in NF-κB activity [53,54]. Two other IκB isoforms (IκBβ and IκBε) are also included in the model. They act in the same way as IκBα, but their production is not modelled as NF-κB-dependent and their activity is in antiphase with IκBα activity. This acts to dampen NF-κB oscillations.

1. Schumann RR, Leong SR, Flaggs GW, Gray PW, Wright SD, et al. (1990) Structure and function of lipopolysaccharide binding protein. Science (New York, NY) 249: 1429–1431.

2. Wright SD, Ramos RA, Tobias PS, Ulevitch RJ, Mathison JC (1990) CD14, a receptor for complexes of lipopolysaccharide (LPS) and LPS binding protein. Science (New York, NY) 249: 1431–1433.

3. Poltorak A, He X, Smirnova I, Liu MY, Van Huffel C, et al. (1998) Defective LPS signaling in C3H/HeJ and C57BL/10ScCr mice: mutations in Tlr4 gene. Science (New York, NY) 282: 2085–2088.

4. Hoshino K, Takeuchi O, Kawai T, Sanjo H, Ogawa T, et al. (1999) Cutting edge: Toll-like receptor 4 (TLR4)-deficient mice are hyporesponsive to lipopolysaccharide: evidence for TLR4 as the Lps gene product. Journal of immunology (Baltimore, Md : 1950) 162: 3749–3752.

5. Takeuchi O, Hoshino K, Kawai T, Sanjo H, Takada H, et al. (1999) Differential roles of TLR2 and TLR4 in recognition of gram-negative and gram-positive bacterial cell wall components. Immunity 11: 443–451.

6. Fenton MJ, Golenbock DT (1998) LPS-binding proteins and receptors. Journal of leukocyte biology 64: 25–32.

7. Hayashi F, Smith KD, Ozinsky A, Hawn TR, Yi EC, et al. (2001) The innate immune response to bacterial flagellin is mediated by Toll-like receptor 5. Nature 410: 1099–1103. doi:10.1038/35074106.

8. Hemmi H, Kaisho T, Takeuchi O, Sato S, Sanjo H, et al. (2002) Small anti-viral compounds activate immune cells via the TLR7 MyD88-dependent signaling pathway. Nature immunology 3: 196–200. doi:10.1038/ni758.

9. Schnare M, Holt AC, Takeda K, Akira S, Medzhitov R (2000) Recognition of CpG DNA is mediated by signaling pathways dependent on the adaptor protein MyD88. Current biology : CB 10: 1139–1142.

10. Häcker H, Vabulas RM, Takeuchi O, Hoshino K, Akira S, et al. (2000) Immune cell activation by bacterial CpG-DNA through myeloid differentiation marker 88 and tumor necrosis factor receptor-associated factor (TRAF)6. The Journal of experimental medicine 192: 595–600.

11. Takeuchi O, Takeda K, Hoshino K, Adachi O, Ogawa T, et al. (2000) Cellular responses to bacterial cell wall components are mediated through MyD88-dependent signaling cascades. International immunology 12: 113–117.

12. Kawai T, Adachi O, Ogawa T, Takeda K, Akira S (1999) Unresponsiveness of MyD88-deficient mice to endotoxin. Immunity 11: 115–122. Available: http://www.ncbi.nlm.nih.gov/pubmed/10435584.

13. Kawai T, Takeuchi O, Fujita T, Inoue J, Mühlradt PF, et al. (2001) Lipopolysaccharide stimulates the MyD88-independent pathway and results in activation of IFN-regulatory factor 3 and the expression of a subset of lipopolysaccharide-inducible genes. Journal of immunology (Baltimore, Md : 1950) 167: 5887–5894.

14. Doyle S, Vaidya S, O’Connell R, Dadgostar H, Dempsey P, et al. (2002) IRF3 mediates a TLR3/TLR4-specific antiviral gene program. Immunity 17: 251–263.

15. Toshchakov V, Jones BW, Perera P-Y, Thomas K, Cody MJ, et al. (2002) TLR4, but not TLR2, mediates IFN-beta-induced STAT1alpha/beta-dependent gene expression in macrophages. Nature immunology 3: 392–398. doi:10.1038/ni774.

16. Hoshino K, Kaisho T, Iwabe T, Takeuchi O, Akira S (2002) Differential involvement of IFN-beta in Toll-like receptor-stimulated dendritic cell activation. International immunology 14: 1225–1231.

17. Lee MS, Kim Y-J (2007) Signaling pathways downstream of pattern-recognition receptors and their cross talk. Annual review of biochemistry 76: 447–480. doi:10.1146/annurev.biochem.76.060605.122847.

18. Yamamoto M, Sato S, Hemmi H, Sanjo H, Uematsu S, et al. (2002) Essential role for TIRAP in activation of the signalling cascade shared by TLR2 and TLR4. Nature 420: 324–329. doi:10.1038/nature01182.

19. Horng T, Barton GM, Flavell RA, Medzhitov R (2002) The adaptor molecule TIRAP provides signalling specificity for Toll-like receptors. Nature 420: 329–333. doi:10.1038/nature01180.

20. Wesche H, Henzel WJ, Shillinglaw W, Li S, Cao Z (1997) MyD88: an adapter that recruits IRAK to the IL-1 receptor complex. Immunity 7: 837–847.

21. Li S, Strelow A, Fontana EJ, Wesche H (2002) IRAK-4: a novel member of the IRAK family with the properties of an IRAK-kinase. Proceedings of the National Academy of Sciences of the United States of America 99: 5567–5572. Available: http://www.pubmedcentral.nih.gov/articlerender.fcgi?artid=122810&tool=pmcentrez&rendertype=abstract.

22. Suzuki N, Suzuki S, Yeh W-C (2002) IRAK-4 as the central TIR signaling mediator in innate immunity. Trends in immunology 23: 503–506.

23. Gottipati S, Rao NL, Fung-Leung W-P (2008) IRAK1: a critical signaling mediator of innate immunity. Cellular signalling 20: 269–276. doi:10.1016/j.cellsig.2007.08.009.

24. Ross K, Yang L, Dower S, Volpe F, Guesdon F (2002) Identification of threonine 66 as a functionally critical residue of the interleukin-1 receptor-associated kinase. The Journal of biological chemistry 277: 37414–37421. doi:10.1074/jbc.M205160200.

25. Muroi M, Tanamoto K (2008) TRAF6 distinctively mediates MyD88- and IRAK-1-induced activation of NF-kappaB. Journal of leukocyte biology 83: 702–707. doi:10.1189/jlb.0907629.

26. Dauphinee SM, Karsan A (2006) Lipopolysaccharide signaling in endothelial cells. Laboratory investigation; a journal of technical methods and pathology 86: 9–22. doi:10.1038/labinvest.3700366.

27. Kollewe C, Mackensen A-C, Neumann D, Knop J, Cao P, et al. (2004) Sequential autophosphorylation steps in the interleukin-1 receptor-associated kinase-1 regulate its availability as an adapter in interleukin-1 signaling. The Journal of biological chemistry 279: 5227–5236. doi:10.1074/jbc.M309251200.

28. Jiang Z, Ninomiya-Tsuji J, Qian Y, Matsumoto K, Li X (2002) Interleukin-1 (IL-1) receptor-associated kinase-dependent IL-1-induced signaling complexes phosphorylate TAK1 and TAB2 at the plasma membrane and activate TAK1 in the cytosol. Molecular and cellular biology 22: 7158–7167.

29. Yamin TT, Miller DK (1997) The interleukin-1 receptor-associated kinase is degraded by proteasomes following its phosphorylation. The Journal of biological chemistry 272: 21540–21547.

30. Irie T, Muta T, Takeshige K (2000) TAK1 mediates an activation signal from toll-like receptor(s) to nuclear factor-kappaB in lipopolysaccharide-stimulated macrophages. FEBS letters 467: 160–164.

31. Wang C, Deng L, Hong M, Akkaraju GR, Inoue J, et al. (2001) TAK1 is a ubiquitin-dependent kinase of MKK and IKK. Nature 412: 346–351. Available: http://www.ncbi.nlm.nih.gov/pubmed/11460167.

32. Arch RH, Gedrich RW, Thompson CB (1998) Tumor necrosis factor receptor-associated factors (TRAFs)--a family of adapter proteins that regulates life and death. Genes & development 12: 2821–2830.

33. Adhikari A, Xu M, Chen ZJ (2007) Ubiquitin-mediated activation of TAK1 and IKK. Oncogene 26: 3214–3226. doi:10.1038/sj.onc.1210413.

34. Covert MW, Leung TH, Gaston JE, Baltimore D (2005) Achieving stability of lipopolysaccharide-induced NF-kappaB activation. Science (New York, NY) 309: 1854–1857. Available: http://www.ncbi.nlm.nih.gov/pubmed/16166516. Accessed 23 July 2012.

35. Yamamoto M, Sato S, Mori K, Hoshino K, Takeuchi O, et al. (2002) Cutting edge: a novel Toll/IL-1 receptor domain-containing adapter that preferentially activates the IFN-beta promoter in the Toll-like receptor signaling. Journal of immunology (Baltimore, Md : 1950) 169: 6668–6672.

36. Yamamoto M, Sato S, Hemmi H, Uematsu S, Hoshino K, et al. (2003) TRAM is specifically involved in the Toll-like receptor 4-mediated MyD88-independent signaling pathway. Nature immunology 4: 1144–1150. doi:10.1038/ni986.

37. Fitzgerald KA, Rowe DC, Golenbock DT (2004) Endotoxin recognition and signal transduction by the TLR4/MD2-complex. Microbes and infection / Institut Pasteur 6: 1361–1367. doi:10.1016/j.micinf.2004.08.015.

38. Yamamoto M, Sato S, Hemmi H, Hoshino K, Kaisho T, et al. (2003) Role of adaptor TRIF in the MyD88-independent toll-like receptor signaling pathway. Science (New York, NY) 301: 640–643. doi:10.1126/science.1087262.

39. Fitzgerald KA, McWhirter SM, Faia KL, Rowe DC, Latz E, et al. (2003) IKKepsilon and TBK1 are essential components of the IRF3 signaling pathway. Nature immunology 4: 491–496. doi:10.1038/ni921.

40. Hemmi H, Takeuchi O, Sato S, Yamamoto M, Kaisho T, et al. (2004) The roles of two IkappaB kinase-related kinases in lipopolysaccharide and double stranded RNA signaling and viral infection. The Journal of experimental medicine 199: 1641–1650. doi:10.1084/jem.20040520.

41. Häcker H, Karin M (2006) Regulation and function of IKK and IKK-related kinases. Science’s STKE : signal transduction knowledge environment 2006: re13. doi:10.1126/stke.3572006re13.

42. Mori M, Yoneyama M, Ito T, Takahashi K, Inagaki F, et al. (2004) Identification of Ser-386 of interferon regulatory factor 3 as critical target for inducible phosphorylation that determines activation. The Journal of biological chemistry 279: 9698–9702. doi:10.1074/jbc.M310616200.

43. Stauber GB, Aiyer RA, Aggarwal BB (1988) Human tumor necrosis factor-alpha receptor. Purification by immunoaffinity chromatography and initial characterization. The Journal of biological chemistry 263: 19098–19104.

44. Kelliher MA, Grimm S, Ishida Y, Kuo F, Stanger BZ, et al. (1998) The death domain kinase RIP mediates the TNF-induced NF-kappaB signal. Immunity 8: 297–303.

45. Zhang J, Cado D, Chen A, Kabra NH, Winoto A (1998) Fas-mediated apoptosis and activation-induced T-cell proliferation are defective in mice lacking FADD/Mort1. Nature 392: 296–300. doi:10.1038/32681.

46. Yeh WC, Shahinian A, Speiser D, Kraunus J, Billia F, et al. (1997) Early lethality, functional NF-kappaB activation, and increased sensitivity to TNF-induced cell death in TRAF2-deficient mice. Immunity 7: 715–725.

47. Liu ZG, Hsu H, Goeddel D V, Karin M (1996) Dissection of TNF receptor 1 effector functions: JNK activation is not linked to apoptosis while NF-kappaB activation prevents cell death. Cell 87: 565–576.

48. Ea C-K, Deng L, Xia Z-P, Pineda G, Chen ZJ (2006) Activation of IKK by TNFalpha requires site-specific ubiquitination of RIP1 and polyubiquitin binding by NEMO. Molecular cell 22: 245–257. doi:10.1016/j.molcel.2006.03.026.

49. Gil J, Alcamí J, Esteban M (2000) Activation of NF-kappa B by the dsRNA-dependent protein kinase, PKR involves the I kappa B kinase complex. Oncogene 19: 1369–1378. doi:10.1038/sj.onc.1203448.

50. Brown K, Park S, Kanno T, Franzoso G, Siebenlist U (1993) Mutual regulation of the transcriptional activator NF-kappa B and its inhibitor, I kappa B-alpha. Proceedings of the National Academy of Sciences of the United States of America 90: 2532–2536.

51. Scott ML, Fujita T, Liou HC, Nolan GP, Baltimore D (1993) The p65 subunit of NF-kappa B regulates I kappa B by two distinct mechanisms. Genes & development 7: 1266–1276.

52. Sun SC, Ganchi PA, Ballard DW, Greene WC (1993) NF-kappa B controls expression of inhibitor I kappa B alpha: evidence for an inducible autoregulatory pathway. Science (New York, NY) 259: 1912–1915.

53. Hoffmann A, Levchenko A, Scott ML, Baltimore D (2002) The IkappaB-NF-kappaB signaling module: temporal control and selective gene activation. Science (New York, NY) 298: 1241–1245. Available: http://www.ncbi.nlm.nih.gov/pubmed/12424381. Accessed 8 March 2012.

54. Nelson DE, Ihekwaba a EC, Elliott M, Johnson JR, Gibney C a, et al. (2004) Oscillations in NF-kappaB signaling control the dynamics of gene expression. Science (New York, NY) 306: 704–708. Available: http://www.ncbi.nlm.nih.gov/pubmed/15499023. Accessed 29 February 2012.
